# Non-invasive Vagus Nerve Stimulation as an Adjunct Treatment for Inappropriate Sinus Tachycardia

**DOI:** 10.19102/icrm.2025.16037

**Published:** 2025-03-15

**Authors:** Ashley Houff, Bernard Gros, Svetlana Blitshteyn, Rebecca Guido, David Fries

**Affiliations:** 1 Department of Medicine, College of Medicine, University of Central Florida, Orlando, FL, USA; 2 Department of Neurology, Jacobs School of Medicine and Biomedical Sciences, Buffalo, NY, USA; 3 Department of Medicine, Rochester Regional Health POTS Clinic, Rochester, NY, USA; 4 Department of Medicine, University of Central Florida-Regional Health POTS Clinic, Orlando, FL, USA

**Keywords:** Autonomic dysfunction, dysautonomia, inappropriate sinus tachycardia, vagus nerve stimulation

## Abstract

Inappropriate sinus tachycardia (IST) is a type of cardiovascular autonomic dysfunction (CVAD) that mainly affects young women and has a prevalence of 1%–2%. IST is characterized by a sinus heart rate of >100 bpm at rest with a mean 24-h heart rate of >90 bpm associated with distressing symptoms such as palpitations, dizziness, and syncope. Here, we discuss a case of a 30-year-old woman who presented with complaints of tachycardia and associated symptoms, including dizziness, diaphoresis, and sudden loss of consciousness. The 24-h Holter monitoring was consistent with the diagnosis of IST. The patient had minimal improvement on β-blocker therapy. Due to persistent symptoms consistent with IST, she was started on non-invasive vagal nerve stimulation (n-VNS) therapy. Following 2 months of n-VNS applied twice daily over the carotid artery, the patient noted near-complete relief of her tachycardia and other debilitating symptoms. While n-VNS has recently been reported as a possible treatment for postural orthostatic tachycardia syndrome, another type of CVAD, to the best of our knowledge, this is the first report of low-level n-VNS as a treatment for IST. Our case study highlights the need for further clinical studies on the benefits of n-VNS in treating IST.

## Case presentation

### History of presentation

A 30-year-old woman with a history of systemic lupus erythematous, hypermobile Ehlers–Danlos syndrome, migraine headaches, and asthma presented with complaints of a 15-year history of intermittent racing heart. Five years prior to presentation, her symptoms had significantly worsened with more frequent episodes of tachycardia. She also experienced frequent episodes of dizziness, lightheadedness, diaphoresis, and trouble hearing, which were at times followed by a sudden loss of consciousness upon standing. In the year before presentation, an episode of loss of consciousness was followed by a week of frequent headaches and memory problems. During the last 6 months before presentation, she experienced tachycardia and associated symptoms daily, both while lying down and standing. The patient also reported bilateral lower-extremity edema, difficulty with sleep, decreased appetite, a reduction in functional aerobic capacity, and marked heat intolerance. She denied paroxysmal nocturnal dyspnea, orthopnea, claudication, fever, and chills. Given the persistence of unexplained tachycardia and associated symptoms, she presented to our postural orthostatic tachycardia syndrome (POTS) clinic for further evaluation.

A written informed consent was obtained for the publication of patient descriptions. This case study was approved by the University of Central Florida Institutional Review Board, and the patient was given the opportunity to review the manuscript.

### Investigations

**Figure 1:**
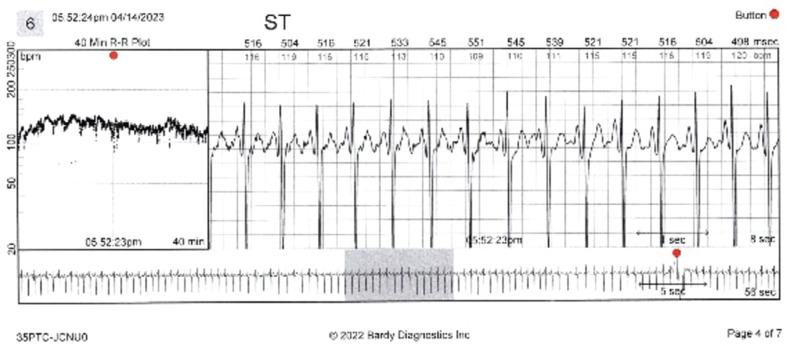
Patient-triggered “event,” depicting sinus tachycardia at rest.

Before starting the treatment, the patient underwent 24-h Holter monitoring, which revealed an average heart rate (HR) of 92 bpm (range, 63–159 bpm) with 37% sinus tachycardia and rare atrial and ventricular ectopy, consistent with the diagnosis of inappropriate sinus tachycardia (IST) **(Figure 1)**.^1^ The average awake HR on her smartwatch was 98 bpm, and the average overall HR was 89 bpm. An echocardiogram demonstrated a structurally normal heart. Her initial electrocardiogram (ECG) revealed sinus tachycardia at 130 bpm. Based on these results, the patient was prescribed metoprolol tartrate.

Upon referral to our POTS specialty clinic, an active stand test was performed while the patient was taking metoprolol tartrate. The 10-min stand test revealed the following HR measurements in bpm and blood pressure (BP) measurements in mmHg **(Table 1)**.

**Table 1:** Active Stand Test Results on Metoprolol Tartrate Before Starting Further Therapy

	HR (bpm)	BP (mmHg)
Supine	89	135/79
Upon standing	95	143/88
After 2 min of standing	92	140/80
After 5 min of standing	88	137/88
After 10 min of standing	90	136/88

Her Holter monitor results, with an average HR of >90 bpm, were consistent with the diagnostic criteria for IST.^1^ The active stand test, albeit potentially confounded by taking metoprolol, did not meet the diagnostic criteria for POTS.^2^ Other causes of sinus tachycardia were also ruled out.

### Management

As ivabradine has been reported as beneficial for IST, the patient’s medication was switched from metoprolol tartrate 12.5 mg twice daily to ivabradine 5 mg twice daily upon referral to our POTS clinic.^1,3^ She noted initial symptomatic improvement, but 4 days after starting the treatment, she experienced a recurrence of persistent tachycardia with a resting HR of up to 150 bpm, shakiness, and feelings of anxiety. Ivabradine was discontinued, and she was switched to metoprolol 12.5 mg every 4 h. While on this regimen, she stated that she felt better but continued to have significant difficulty with orthostatic intolerance, which impacted her ability to work and socialize. Given the persistence of symptoms, and despite ivabradine and metoprolol interventions, the patient was referred from her outpatient cardiologist to our POTS clinic for further evaluation.

She was switched to atenolol 25 mg daily. She was also advised to drink 2–3 L of water daily, consume 6–10 g of sodium as part of her daily diet (supplemented with 1–2 buffered salt tablets each meal), begin daily exercise, and include restorative yoga. She was advised to first start with minimal gravitational stress exercises, such as recumbent bicycling or swimming, before progressing to a rowing machine or stationary bicycle, and then graduate to upright activity. The patient reported improvement upon starting atenolol and non-pharmacologic interventions but continued to have daily 30-min to 8-h episodes of supine tachycardia with an HR of >100 bpm, associated with headache, fatigue, and palpitations.

Due to persistent symptoms consistent with IST, as well as intermittent migraine headaches, she was started on non-invasive vagal nerve stimulation (n-VNS) therapy. Specifically, she used a GammaCore Sapphire n-VNS at a sinusoidal wave, symmetrical biphasic frequency of 5 kHz pulses at a rate of 25 Hz, maximum output 30 V (peak), and a load impedance of 450–550 ohms. The patient applied n-VNS over the left carotid artery for 6 min every morning and the right carotid artery for 6 min every evening. The intensity of the stimulation is titrated by the patient to achieve a tingling in the lips or a slight muscle contraction at the corner of the mouth. Following 2 months of this intervention, the patient noted near-complete relief of her tachycardia symptoms, including her palpitations, many of which were disabling, along with partial improvement of her diminished exercise tolerance and blood pooling symptoms. Her in-office HR and BP were 87 bpm and 129/74, respectively, while sitting. Following combined therapy with a β-blocker and n-VNS, she noted that her tachycardia symptoms occurred only 1–2 times a month for 1–3 days, often coinciding with her menstrual cycle, when previously she was experiencing persistent daily tachycardia (both supine and standing). She contended that n-VNS was most helpful for her orthostatic symptoms, while atenolol provided greater assistance with supine tachycardia.

The patient had previously used n-VNS for the treatment of migraine headaches but was still experiencing inappropriate tachycardia symptoms at that time. She found that the combination of n-VNS with atenolol, however, was associated with a marked improvement in her autonomic symptoms. Her reported mean awake HR during the trial period, as detected by her smartwatch, was 80–85 bpm, with a mean overall HR of 74–78 bpm.

She reported mild neck and throat discomfort while using n-VNS, but denied pain, lasting discomfort, and other side effects. After 5 months of using n-VNS twice daily, she discontinued this treatment due to the cost of this therapy. Following discontinuation and while remaining on atenolol, her symptom relief persisted and has now reached a total duration of symptom relief of 9 months.

## Discussion

IST is a type of cardiovascular autonomic dysfunction (CVAD) that has an estimated prevalence of 1%–2%.^3,4^ CVAD syndromes, including IST and POTS, mainly affect young, premenopausal women.^2,3^ IST is a diagnosis of exclusion characterized by a sinus HR of >100 bpm at rest with a mean 24-h HR of >90 bpm associated with distressing symptoms such as palpitations, dizziness, and syncope.^1,3^

IST is typically initially assessed with a 12-lead ECG followed by 24-h Holter monitoring for diagnosis.^1^ To evaluate for POTS, an active stand test or a formal upright tilt table test may be performed.^2^ Obtaining laboratory values, such as complete blood and thyroid function tests, may also be indicated to rule out other causes of sinus tachycardia.^1^

Current standard treatments begin with the initiation of non-pharmacologic interventions, such as liberal dietary salt and water intake to increase blood volume, limiting caffeine intake, a daily exercise program for physical reconditioning, and wearing compression stockings.^1,5^ For some patients, ivabradine may be more effective than β-blockers, but the cost of ivabradine limits its use.^1^

To the best of our knowledge, low-level n-VNS has not been previously reported as a treatment for IST. However, low-level n-VNS has improved POTS-like symptoms in rabbit models.^6,7^ Specifically, the rabbit models had auto-antibodies to the muscarinic acetylcholine receptor M2, α1-adrenergic receptor, or the β1-adrenergic receptor.^6,7^ Following 2–4 weeks of daily vagal nerve stimulation, the rabbits had decreased postural tachycardia and variability in HR.^6,7^ It is theorized that the increase in acetylcholine from vagus nerve stimulation improved the rabbit POTS symptoms by increasing parasympathetic activity enough to overcome the auto-antibody–induced increased sympathetic activity.^6^

Recently, n-VNS has also been reported as a possible treatment for POTS in humans, though stimulation was applied to the tragus rather than the carotid artery.^8^ As in the rabbit studies, the current understanding is that n-VNS increases parasympathetic activity and therefore decreases the excess postural sympathetic activity in POTS.^9^ Additionally, n-VNS has also been shown to decrease inflammation via reduction of pro-inflammatory cytokines, which may contribute in part to IST found in CVAD.^9^ Lastly, as our patient had lupus, n-VNS may have been effective in her case by reducing inflammation and auto-immunity, which can lead to autonomic dysfunction underlying IST. The existing reports of the effectiveness of n-VNS in relieving postural tachycardia and HR variability in rabbits^6,7^ and humans^8,9^ led us to try n-VNS in our patient with IST.

In this report, we described a patient with significantly improved IST with the use of n-VNS applied to the carotid area. Her symptoms remained improved after discontinuing n-VNS therapy, making it difficult to attribute the improvement to n-VNS alone. However, our patient experienced both a subjective improvement in symptoms and a documented objective reduction in resting HR with n-VNS, suggesting possible n-VNS contribution to parasympathetic nervous system enhancement and autonomic recovery. A placebo effect cannot be ruled out given the limitations of a single case report. Therefore, future research is needed to determine the effectiveness of adding n-VNS to treatment regimens compared to the current standard non-pharmacologic and pharmacologic treatments for IST.
